# Policy and practices shaping the delivery of health services to pregnant adolescents in informal urban settlements in Kenya

**DOI:** 10.1093/heapol/czad070

**Published:** 2023-11-16

**Authors:** Linet Okoth, Rosie Steege, Anne Ngunjiri, Sally Theobald, Lilian Otiso

**Affiliations:** LVCT Health, Nairobi, Kenya; Liverpool School of Tropical Medicine, Liverpool, United Kingdom; MARCH Centre, London School of Hygiene and Tropical Medicine, London, United Kingdom; LVCT Health, Nairobi, Kenya; Liverpool School of Tropical Medicine, Liverpool, United Kingdom; LVCT Health, Nairobi, Kenya

**Keywords:** Pregnant adolescents, informal urban settlement, Kenya, reproductive justice, sexual reproductive health services

## Abstract

In Kenya, the pregnancy rate of 15% among adolescents aged 15–19 years is alarmingly high. Adolescent girls living in informal urban settlements are exposed to rapid socio-economic transitions and multiple intersecting health risks and may be particularly disadvantaged in accessing sexual reproductive health services. Understanding vulnerabilities and service-seeking behaviours from different perspectives is important in order to support the development and implementation of progressive policies and services that meet adolescents’ unique needs within urban informal settlements. This study explored policy makers, community health service providers’ and community members’ perceptions of access to, and delivery of, sexual reproductive health services for pregnant adolescents in one informal urban settlement in Nairobi. We employed qualitative methods with respondents throughout the health system, purposively sampled by gender and diversity of roles. We conducted focus group discussions with community members (*n* = 2 female-only; *n* = 2 male-only), key informant interviews with policy makers (*n* = 8), traditional birth attendants (*n* = 12), community health volunteers (CHVs) (*n* = 11), a nutritionist (*n* = 1), social workers (*n* = 2) and clinical officers (*n* = 2). We analysed the data using thematic analysis. Government policies and strategies on sexual and reproductive health for adolescents exist in Kenya and there are examples of innovative and inclusive practice within facilities. Key factors that support the provision of services to pregnant adolescents include devolved governance, and effective collaboration and partnerships, including with CHVs. However, inadequate financing and medical supplies, human resource shortages and stigmatizing attitudes from health providers and communities, mean that pregnant adolescents from informal urban settlements often miss out on critical services. The provision of quality, youth-friendly reproductive health services for this group requires policies and practice that seek to achieve reproductive justice through centring the needs and realities of pregnant adolescents, acknowledging the complex and intersecting social inequities they face.

Key messages

- Analysis of health systems is important to assess how their building blocks and actors act, react and interact with each other to influence adolescent health service utilization and delivery in informal urban contexts in Nairobi.
- Despite a conducive policy environment on adolescent sexual and reproductive health and innovative practice at some health facilities, policies too often evaporate in practice within the devolved Kenyan context.
- The broader environment and intersecting equities shape pregnant adolescent’s health-seeking journeys; reproductive justice requires centring in order to respond to adolescents’ needs within their social context.

## Introduction

Despite a decline in the Kenyan national fertility rate, adolescent fertility rates are not declining at the same rate (George *et al.*, 2021). According to the recently launched 2022 Kenya Demographic and Health Survey, the pregnancy rate among adolescents aged 15–19 years stands at 15% (KNBS, MoH *et al*., 2023). On contraceptive use, a direct determinant of pregnancy, almost three in five (59.3%) of all sexually active adolescents aged 15–19 years who identify as currently married females are not using any method of contraception, and two in five (41.6%) of their unmarried counterparts do not use contraceptives (KNBS, MoH *et al*., 2023). Adolescent pregnancy is a major public health concern since it increases the risk of maternal and neonatal mortality and morbidities. Adolescent pregnancies are associated with poor birth outcomes and have long-term implications for quality of life (Diabelková *et al.*, 2023). For example, obstetric fistulas can leave girls incontinent and at risk of unemployment, depression, social isolation and stigma (Todhunter *et al.*, 2022). Poor social or family support, unstable relationships, and inadequate access to health facilities or to educational opportunities push adolescents into psychiatric and health-related morbidities with poorer outcomes for their children contributing to a cycle of vulnerability (Patton *et al.*, 2016; KNBS, MoH *et al*., 2023).

Adolescents in Kenya face significant interconnected challenges that increase their vulnerability to early pregnancies, and barriers to accessing sexual and reproductive health (SRH) services. These obstacles may include poverty, a lack of comprehensive information about contraception, limited access to sexual and reproductive health care, high vulnerability to sexual violence and inadequate parental support (Mumah *et al.*, 2020). These challenges may be particularly acute for adolescents living in urban informal settlements (Gillespie *et al.*, 2022). Informal settlements are characterized by a lack of basic social infrastructure, overcrowding, a lack of tenure security and high health-care costs impeding access to health services (Fotso *et al.*, 2008; Kimani *et al.*, 2021). The structural inequalities facing adolescents living in informal urban settlements can also be a determinant of risky sexual behaviour. These risks include: early sexual debut, ‘transactional’ sex and having multiple sexual partnerships (Mumah *et al.*, 2020). Statistics show that adolescents from poorer backgrounds are three times more likely to have begun child bearing compared to those from relatively wealthier backgrounds (KNBS, MoH *et al*., 2023). Once pregnant, factors impeding access to appropriate, quality maternal care for adolescents include: poor health education, the inability to afford costs of pregnancy and childbirth, health-care workers’ negative attitudes, and limited implementation of policies focused on the provision of SRH services to adolescents (MoH Kenya, 2015; Rosen *et al.*, 2015; Erasmus *et al.*, 2020; Mumah *et al.*, 2020; Mutea *et al.*, 2020).

The Kenyan government recognizes that access to SRH services for adolescents is a fundamental right and seeks to support adolescent-friendly health services as a national priority (MoH Kenya, 2015; George *et al.*, 2021). The government of Kenya (GoK) has therefore rolled out several policies targetted at adolescent health in Kenya. These include: the Health Act 2017 (GoK, 2017), which identifies health service provision as a fundamental duty of the State to observe, respect, protect, promote and fulfil the right of the population to the highest attainable standard of health including reproductive health care and emergency medical treatment; the Adolescent Sexual and Reproductive Health (ASRH) policy (MoH Kenya, 2015), which promotes ASRH services; and the Constitution of Kenya 2010 (GoK, 2010), which highlights health as a right for all Kenya citizens. This last policy underlines free maternity care as a key strategy to support access to services for all women of reproductive age—including adolescents, known as ‘Linda Mama’ (Orangi *et al.*, 2021). The National Guidelines for School Re-entry in Early Learning and Basic Education also outlines a school re-entry guidance on attracting girls back to school during pregnancy and after delivery (MoH Kenya, 2020). Nairobi County, following devolution in 2013, identified adolescent health as a priority group in the health system and widely disseminated these policy documents to improve access to SRH services (MoH Kenya, 2017). Yet, there has been very little research in order to gain an insight into the barriers and enablers of policy implementation for this group, particularly within urban informal settlements. Understanding how policies are implemented and enacted within the context of urban informality is critical, given the multiple factors that exacerbate risk of early sexual debut, adolescent pregnancy, and limited uptake of sexual and reproductive health services (Green *et al.*, 2017). Understanding these policies will support reproductive justice for this group by informing responsive policies that are grounded in contexual realities.

This research study sought to explore the unique circumstances that influence policy implementation, as well as access to and utilization of SRH services for pregnant and parenting adolescent girls (PPAs) living in Korogocho urban informal settlement. We have mapped the barriers and enablers in adolescent-friendly SRH policy implementation across the health system and have drawn out the interlinkages and key social and relational factors that influence policy implementation, situating the health system within the context of informality. We have captured the perspectives of SRH health-care providers (HCPs) within the health system, policy makers, and the broader community in order to provide recommendations to improve maternal health service delivery to PPAs. Data collected directly from PPAs will be reported in a separate publication.

## Methods

### Study design

We employed an exploratory qualitative study design.

#### Setting

The study was conducted in Korogocho informal settlement, Nairobi’s fourth largest informal urban settlement. Korogocho is congested with 150 000–200 000 people living in an area of 1.5 square kilometres, a stable and settled population, with many long-term residents (Emina *et al.*, 2011). There is high unemployment and extensive violence and insecurity. Residents are young, highly mobile, have varied ethnicity and live close to employment opportunities. Korogocho comprises structures mostly made of mud and timber walls with waste tin cans as roofing material (Beguy *et al.*, 2015). It has limited basic infrastructure and social amenities, such as roads, educational, health and sanitary facilities, and recreational areas. The poor living conditions predispose residents to severe health problems, aggravated by the scarce availability and accessibility of health facilities. Young people, who constitute a third of Korogocho, face unique challenges as they transition to adulthood, including poor schooling outcomes, early marriage, illiteracy, sexual and gender-based violence and unintended pregnancies (Beguy *et al*., 2012; Wamukoya *et al.*, 2020).

#### Study participants

Health-care providers including clinicians, nurses, medical social workers, a nutritionist, traditional birth attendants (TBAs) and community health volunteers (CHVs) were engaged in the study. We also engaged policy- and decision makers at national and sub-national levels who serve as technical experts in maternal, SRH, adolescent and child health programming. To obtain a holistic view on our research question, we also recruited male and female community members who were parents or caregivers of adolescents to share their opinion on PPAs and access to maternal health services.

#### Inclusion and exclusion criteria

Participants/informants were eligible if they: (1) were willing to participate and provided written consent, (2) were aged over 18 years, (3) agreed to be audio recorded and have notes taken during data collection, and (4) were conversant in either English or Kiswahili. HCPs must have worked at the Korogocho Health Centre. TBAs were eligible if they were currently involved in maternal care to PPAs, and community members must have resided in the Korogocho for over 12 months, and be parents or caregivers of adolescent girls.

#### Recruitment of participants

Proposed HCP participants were recruited from the local health facility within the study site. They were purposively sampled based on their role in the provision of adolescent SRH services. CHVs were purposively sampled from the Community Health Unit that serves the Korogocho ward and referred by the community health assistant (MoH Kenya, 2020). CHVs helped to identify TBAs, who identified further respondents. Community members were purposively sampled based on recommendations by CHVs. Policy makers were identified from reproductive, maternal, child and adolescent health technical working groups. Participants were contacted by phone, letter or email and provided with information on the purpose and method of the research before giving informed consent to participate. Efforts were made to ensure diversity of gender and variety of roles linked to adolescent SRH in the community, facility or at policy level.

#### Data collection methods

Data was collected in July 2021. Focus group discussions (FGDs), in-depth interviews (IDIs) and key informant interviews (KIIs) were conducted using semi-structured guides, as described in Table 1 below.

**Table 1.** Description of study objective, data collection tools and study participant

Sub-objective	Data collection methods	Participant/informant and sample size
To explore health and social care providers’ perspectives on challenges of access and delivery of maternal health services to pregnant and parenting adolescents and ways to improve these	IDIs with direct MNCH HCPs at community and facility level within the locality of the study area	Social worker (*n* = 2) Clinician (*n* = 2) CHV (*n* = 11) Nurse (*n* = 9) Nutritionist (*n* = 1) TBA (*n* = 12)
To understand policy makers’ perceptions of accountability structures for maternal health services for pregnant and parenting adolescents and ways to improve these	KIIs with influencers/ decision makers who influence MNCH service delivery	A representative from the national level (*n* = 1), county level (*n* = 3) and sub-county level (*n* = 4) from relevant ministries with technical expertise in programming for MNCH, sexual and reproductive health, sexual and gender-based violence, children, adolescents and youth
To explore communities’ perceptions of challenges and enablers to accessing and utilizing maternal health-care services for pregnant adolescents in Korogocho	FGDs with community members in Korogocho	Male-only FGD with 8 participants each (*n* = 2) Female-only FGD with 8 participants each (*n* = 2)

CHV, community health volunteers; FGD, focus group discussions; HCP, health-care providers; IDI, in-depth interviews; KII, key informant interviews; MNCH, maternal, newborn and child health; TBA, traditional birth attendants.

The IDI and FGD topic guides were developed in English, translated into Kiswahili and reviewed for consistency. The guides were pilot-tested in another urban informal settlement outside the study area among individuals with similar characteristics.

#### Fieldwork data collection

Data were collected by four trained research assistants (two male, two female), who had received a prior four-day training on ethics, data collection tools and key techniques on qualitative data collection and participant engagement. An experienced moderator and note-taker led the FGDs; each FGD had eight participants and was separated by sex. The FGDs and IDIs were carried out in both English and Kiswahili languages, in a community space conducive to the exercise. Confidentiality was observed throughout. All except two interviews were conducted face-to-face. One KII was conducted through a recorded telephone conversation, and another via a Zoom call. For those interviewed virtually, scanned consent forms were sent by email and were password-protected. Conversations were audio-recorded and field notes were taken. Data was collected until thematic saturation was reached.

### Data analysis

The audio recordings were transcribed verbatim into English (from Kiswahili) and uploaded to NVivo Version 12 software. Three experienced research team members coded the transcripts independently using a jointly developed code book. Emerging themes not anticipated were added to the study guide and the dimensions followed up in the subsequent interviews. A final thematic framework was developed based on inductive and deductive themes to support coding and analysis (Ritchie and Spencer, 2002).

### Ethical consideration

Ethical approval was obtained from the United Kingdom [LSTM IRB Protocol (21–007) ‘Accountability for Informal Urban Equity Hub (ARISE)’] and in Kenya [AMREF Ethics & Scientific Review Committee (ESRC), ESRC P747/2019 as part of the broader ARISE study]. We also gained approval from the National Commission for Science, Technology and Innovation (NACOSTI), Kenya for the study and consent from Nairobi County. Informed consent was obtained for all respondents.

## Results

Table 2 presents the demographic and occupational characteristics of the study participants. A total of 37 IDIs, 4 FGDs and 9 KIIs were conducted.

**Table 2.** Demographic characteristics of the study participants

	Sex		Years of occupation		
	Male	Female	<1 year	2–5 years	>5 years
CHV (*n* = 11)	3	8	1	3	8
TBA (*n* = 12)		12			12
Clinical officer (*n* = 2)	1	1	1	1	
Social worker (*n*= 2)	1	1		1	1
Nutritionist (*n* = 1)		1		1	
Nurse (*n* = 9)	1	8	2	4	3
Community member (*n* = 32)	16	16			
Policy maker (*n* = 8)	1	7	2	2	4

Our findings are structured around the key building blocks described in the World Health Organization (WHO) health systems framework (Sameen Siddiqi *et al.*, 2022) that influence the policy implementation of SRH services for pregnant adolescents.

## Governance

### Availability of appropriate policies on adolescent and youth sexual reproductive health

The policy- and decision makers interviewed explained that there are many policies that address adolescent SRH in Kenya, and they articulate the right of access to free maternal and SRH services in public health facilities to include private providers through the National Hospital Insurance Fund (NHIF). The National Adolescent Sexual Reproductive Health policy 2015 and the National Guidelines for School Re-Entry in Early Learning and Basic Education (Government of Kenya, 2020) support the re-integration of adolescents post-partum to continue their education. This was seen by policy makers as an important and holistic strategy:

… *they’ll be re-socialised and taken back to school, and in terms of medical care they’ll attend all the clinics up to delivery, and even delivery …* (KII, County policy maker 1)

### Partner coordination and partnerships

The County government promotes the establishment of youth-friendly spaces as a strategy to improve access to SRH services for young people. This is done by working with partners and management at the health facility to identify, set up and equip these spaces with appropriate SRH information, education for young people and the provision of essential SRH goods and services. The County also works with other Ministry of Health partners, e.g. USAID-funded mechanisms in the County to train and deploy young service providers, to encourage relatability.

As part of joint governance, policy makers at the Ministry of Education reported being involved in technical working groups that encourage joint efforts in adolescent health service provision. However, services like counselling that could not be offered in their department were outsourced through referral.

*We mainly do referral since we do not have key policies in our department to offer counselling and so we have to coordinate with the other departments…* (Sub-County policy maker 1)

The County has many health partners implementing different interventions that target adolescent SRH. Participants reported that the County liaises with its partners providing periodic reviews and status updates to ensure links with County SRH priorities and to avoid duplication and competition. This has expanded the coverage for young people while promoting effective resource use.

### Impact of devolution

Devolution of the health sector in 2013 allowed counties to develop their own policies and implementation strategies. The education sector participants recognized the importance of encouraging parents and caregivers to discuss sexual and reproductive health with their adolescents:

*We do a lot of capacity building in terms of parenting skills so we use those forums to sensitise parents and caregivers and even opinion leaders within the community on issues of girl child especially when it comes to sex education, yeah*. (KII, County education sector policy maker 2)

This statement highlights the benefit of having autonomy to adapt policies and training at the County level, which is a result of devolution. This autonomy has also been extended to health facility management. During their interviews, managers at the facility reported how continuous feedback mechanisms are used to improve services and that age and sex disaggregated data is used to support policy development for adolescents illustrating how strengthened information systems are within the health systems blocks.

*The facilities are able to use data from Kenya Health Information System (KHIS) segregated by age and use it for decision making*. (County policy maker 3)

## Service delivery

### Quality of service delivery in health facilities

Interviews with nurses at the facility highlighted how facilities were already overstretched due to a lack of equipment and staff to provide quality services in a timely manner. Bed capacity is reportedly low and the facilities are ill-equipped to provide quality services and meet demand:

*…lacking the commodities, like a teenage girl comes for the antenatal clinic and they are supposed to be given the ANC books, they are supposed to be given supplements—[these are] missing. For family planning you find some methods and commodities are not there, so it becomes quite a challenge to offer them because they might come and want this and that method but it is not there, sending them to another facility and they won’t go, so they will go at home and wait, wait till we have them.* (IDI, health worker 3)

To improve service quality for PPAs and adolescents in general, health facility staff implemented continuous improvement initiatives to support adolescent-friendly services, such as:

- Adjusted opening hours for services for young people to enable them to access services after school and work.
- Dedicated staff to attend to adolescents’ SRH needs in a youth-friendly manner. This also means that adolescents do not have to queue for many hours to receive services and reduces their interaction with people they perceive as judgemental, serving to reduce discrimination.
- Allowing mentors and CHVs to identify and triage adolescents and direct them to the relevant youth responsive service delivery points, increasing effective referral.

*In most cases they fear so much and they feel ashamed so somebody can conceal her problem. So when we go round by good luck, we find them. So, if we find them, we direct and help them and they go to the hospital and receive treatment. You find that those nurses who are there know how to handle them.* (FGD, community health volunteer 1)

- Express services given to women accompanied by their male partners (including the pregnant adolescents) to encourage male/partner involvement in service utilization. This innovation, however, can end up discriminating against adolescents as their male partners are often their peers or older men, who risk persecution for impregnating an adolescent and are uncomfortable to join them to access services.
- The facilities also arranged for health talks on topical issues during clinic days for women and girls especially touching on SRH issues.

## Financing and medical supplies

### Lack of adequate financing and resources

Policy makers interviewed reported an increased demand for all services including SRH services in the hospitals, although no additional funding has been provided by the County. Policy makers confirmed that periodic stockouts of essential medical commodities and equipment was a barrier to users accessing SRH services. They also reported that stockouts were caused by limited budgets, late disbursement of funds for purchase of essential commodities, and long supply chains. This resulted in pregnant women not receiving adequate services from health facilities when they needed them, and informal payments for commodities, leading to reduced demand and poor confidence in health services. Further, it was reported that some HCPs at the facilities ask for additional (informal) financial renumeration to offer SRH services, which further damages confidence in the health system.

*I am always told there is no medicine, there are no reagents, always there is nothing, you know that is now politics. I go to the community and start telling people there is always no treatment being offered here, the government-led institutions have no benefits, when a young child gets to hear that, even if you tell them to come for clinic here they will not come because she heard you talk about how bad it is.* (FGD, community member 2)

*The barriers; we don’t have the necessary (commodities)… what we need for now, when they come maybe we tell them to go and buy drugs and they cannot afford.* (IDI, health-care worker 1)

Despite the free maternal services offered at the facilities, adolescents may face other direct and indirect costs that limit their access—time away from school, fare to the health facility, and the cost of buying unavailable commodities such as cotton wool and gloves, and scans and the cost of fuel for the ambulance during referral in case of an emergency. This may be a particularly limiting factor for adolescents living in urban informal settlements.

… *provision of services like an ultrasound, which is expensive and is preferred from government facilities. Lack of money for services need deters adolescent girls from seeking ANC services till they are due.* (IDI, health-care worker 4)

A lack of resources has also affected the establishment of the youth-friendly spaces defined in the policy for providing responsive programmes to adolescents.

*Challenges again in the facility is space, so I cannot say we have special rooms where we attend to the youths…*. (KII, Sub-County policy maker 2)

## Human resources

Service providers are trained and equipped to provide integrated adolescent SRH information and services whether in the facility, or during outreach. These services include support for safe delivery and post-delivery recovery. The nurses who are trained and knowledgeable about adolescent service provision are able to interact with users and provide the services. Those that have not been trained requested capacity-building to handle adolescents:

*You find that those nurses who are there knows how to handle them….*(KII, Sub-County policy maker 3)

… *we need to be empowered and learn what is new in managing the teens*. (IDI, health worker 1)

To address programme quality, the County health service staff provide support supervision to the health facilities. Gaps in providing quality SRH services, including inadequate knowledge and skills in handling adolescents during service provision, were identified by the health facility staff through interrogation of their own data summaries, which are collated monthly. These gaps were then reportedly addressed through on-the-job training and continuous medical education and capacity-strengthening of health-care workers on issues critical to delivery of quality health care for adolescents.

… *there is a lot of mentorship, there is coaching which is continuous and also as the Sub County team, we do continuous supportive supervision yes*. (KII, Sub-County policy maker 4)

### Health workers’ knowledge and attitudes

Despite the existence of adolescent-appropriate policies, interviews with nurses at the facility revealed little knowledge about these policies among health-care providers, thus limiting implementation. Health-care providers have not been comprehensively trained on the policies and as a result cannot use them as a basis to improve service delivery, creating quality and access barriers. For example, the MoH allows provision of SRH to a mature/emancipated minor (an adolescent who is married, pregnant, sexually active or believed to be at risk of acquiring HIV) without parental consent. However, many care providers requested parental consent to receive services for those under the age of 18 years in line with the 2010 Kenyan Constitution and the Sexual Offences Act. These nuances in the guidance reportedly resulted in confusion among service providers who then decline service provision. This results in missed opportunities for adolescents to receive care and support because of different interpretations of policies.

Some health-care workers were reported to be rude, putting pregnant adolescents off accessing services at the health facilities, highlighting how health systems are social institutions and can reflect and reinforce harmful narratives around adolescent pregnancy.

*With all the health-care providers who are not capacity built, they‘re not able to handle sexual reproductive health issues for the adolescent and the young people. They might find a care provider who doesn’t understand the young people. So, attitude issues come in*. (KII, Sub-County policy maker 6)

### Health-worker safety concerns

One manager noted concerns for the safety of health-care workers while working in the informal settlement due to the high insecurity. This affects service provision at the facility and outreach services in the community.

*[S]ometimes the community is not welcoming, yeah you cannot go to the community, inside the community because of history of robberies, so we have heard that this place is not good*. (IDI, health-care worker)

### Use of traditional birth attendants

In this study TBAs were reported to provide an enabling environment for adolescents in spite of their role in delivery being banned by the MoH. Unlike health-care workers, TBAs offer incentives such as massage and hot tea after delivery, which was reported to be a motivating factor for adolescents to use their services. This may be particularly important in urban informal settlements where poverty is high, and the provision of a meal is valued.

*There are many traditional birth attendants here, like where I live there is this elderly mother who mothers usually come to and she massages them… .* (FGD, community member 1)

… *so the fear the girls have is after giving birth they have to get someone to go buy them food. When you give birth in the house, tea is available, you will get lunch and before you leave in the evening, I will make you tea.* (IDI, traditional birth attendant 1)

## Context and interrelated social factors

### Informal settlements

Community members reported that pregnant adolescents are referred to as ‘adult women’ by the community and that their parents may expect them to fend for themselves. CHVs interviewed reported that the pregnant girls are sometimes chased away from home leading to physical abuse and blackmail from the parents and society; this limits their opportunity to access SRH services, and increases cases of single parents and abandoned children in the urban informal settlement. Being young, they are vulnerable to trauma and other mental health problems, and one CHV highlighted that the pregnancy can also be the result of gender-based violence, which is prevalent in the setting:

… *back at their home their mother does not have money… does not know that the daughter is pregnant, sometimes maybe she is also a cruel mother … whoever impregnated her is a neighbour, or her father or the young school boys.* (IDI, community health volunteer 2)

### Stigma and discrimination

There is a lot of stigma attached to adolescent pregnancy, which results in shame and fear of accessing health services. Community members and TBAs reported that some cases of adolescent pregnancy in the community can lead to unsafe abortion and even suicide. Deaths may also occur from unsafe abortion, or long-term reproductive health complications such as the loss of their womb. CHVs and reformed TBAs play a key role in educating adolescents to ensure that pregnant adolescents receive quality health services and address stigma and discrimination among the adolescents and community at large. CHVs are able to identify, refer and follow up pregnant adolescents as highlighted in the community strategy policy document (MoH Kenya, 2020).

*After knowing that a certain girl is pregnant, you just go to her and talk to her about considering starting going for her clinic early enough, not to feel ashamed and not to be afraid so, you just talk to her and look for the CHVs and tell them to hold them and show them how they should go for clinic.* (FGD, community member 1)

… *one was sent away by the parent and wanted to commit suicide, we told her getting pregnant isn’t the end of the world, she’ll get pregnant, give birth and go back to school.* (IDI, traditional birth attendant 2)

The County has also taken the initiative to address some of the social factors including stigma facing adolescent girls who may have been rejected by their families. This was reportedly done by engaging with various stakeholders to ensure they are provided a shelter and other services required to ensure a successful pregnancy.

… *we have specific donors who support them. We have regulations that guides and promote the operations, we have these girls who have been rescued and taken to these safe houses. We have special services that they get.* (KII, County policy maker 7)

### Cultural and religious sensitivity

According to the policy makers, certain cultures and religions promote practices that inhibit access to SRH for young people. This is a restriction in our policies and not necessary a religious issue but it comes from our cultural and religious practices. An example is age restrictions for adolescents at primary schools (ages 10 to 14 years) to receive sexual education—including information on access to SRH. Some religious beliefs exclude contraception use, leaving sexually active adolescents exposed to pregnancies and sexually transmitted diseases. The religious culture also leaves them unable to access services once pregnant due to stigma and discrimination, depriving them of potential support circles.

*As a Catholic myself, the moment I will be pregnant, I will be afraid to go for clinic because, church people will know I got pregnant before I was married so, you see I have already put a barrier and I will not even go to church because, I am not married and I am ashamed of myself so, it is also a contributing factor, it will prevent you from going to church so that, church members will also not realise that I am pregnant.* (FGD, female participant 1)

*You find for other religious… Maybe you find a place that has a lot of Muslims, they will prefer not to talk about that at the age of 10; they want to talk about it later when the children are a bit grown, maybe in high school. Again, I think culture, beliefs of the people, different people have different beliefs; some believe it is too early.* (KII, Sub-County policy maker 5)

## Discussion

### Summary of findings

**Figure 1.**
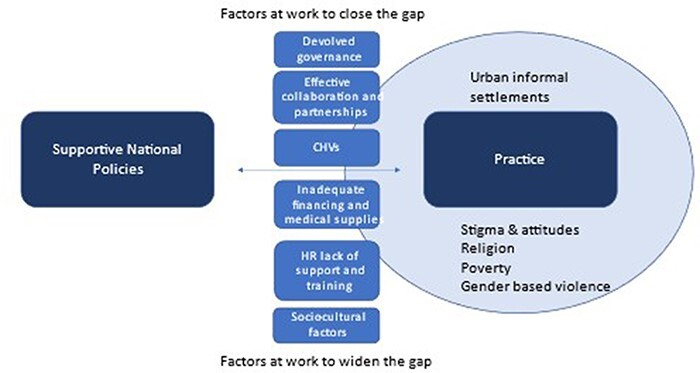
Delivery of health services to pregnant adolescents in informal urban settlements

We triangulated the views of policy makers, health service providers and community members against five key WHO health systems building blocks (governance, finance, essential supplies, human resources for health, and service delivery) and situated our findings within the context of informal settlements and the social, cultural and religious sensitivities surrounding adolescent pregnancies. As shown in Fig. 1 we found that there are supportive national government policies and strategies on sexual reproductive health of adolescents and young people, and positive enablers from the devolved context. However, challenges remain to the effective implementation of policy in practice within the contexts of informal settlements in Nairobi. Key factors contributing to this include the inadequate financing and medical supplies, the lack of consistent supply of essential commodities or equipment leading to perceived poor quality services, additional costs and community members seeking alternative services. This was compounded by a lack of human resource training, disrespectful attitudes of health providers shaped by socio-cultural attitudes around adolescent pregnancy, confusion over policies and legal frameworks, including a legal framework that does not support the inclusion of male partners, and safety fears of health-care workers in urban informal settlements.

Our findings demonstrate how effective leadership and governance are not sufficient alone to support the implementation of adolescent-friendly services in the context of urban informality in Nairobi. The adolescents come from a low social economic status that exposes them to varied challenges including gender-based violence, neglect and discrimination from their peers, family members, partners and even formal health-care workers because of their pregnancy. These cross-cutting factors limit the implementation of well-intentioned policies, which are discussed below.

### Benefits of devolution and key gaps

Devolution in Kenya began in 2013 resulting in a division of roles among different levels in the health system, distributing power that was previously at the national level to counties. The national level provides policy and guideline formulation whereas the counties (districts and sub-districts) execute the policies from county to community level (McCollum *et al.*, 2018). Policy makers at national level reported availability of policies that guide the delivery of adolescent sexual and reproductive services; however, service providers at health facilities and community volunteers strongly felt that the policies were not disseminated adequately to support transfer of skills and knowledge. Thorough training that considers the social and political aspects that influence service utilization to ensure that all service providers understand the importance of youth-friendly and person-centred services is necessary to reap the dividends of the policies (Embleton *et al.*, 2023). This calls for adequate financing, human resources, training and supportive supervision in the counties to be provided as a comprehensive package that embraces the autonomy that came with devolution.

Our findings support a recent analysis by the Global Financing Facility (GFF) (an initiative hosted by the World Bank that aims to increase financing for women’s, children’s and adolescent’s health). The GFF analysis demonstrated that although adolescents are reflected well in the national investment case, this commitment was not converted into indicators or financial commitments (George *et al.*, 2021). According to the National and County Health Budget Analysis 2020/21 (MoH Kenya, 2021), Kenya has made ‘tremendous strides’ in keeping up with the public health financing commitment made in the Abuja Declaration of 2001 (Embleton *et al.*, 2023). The declaration proposed that member states allocate 15% of their domestic budget to support health as countries moved towards self- reliance (substituting domestic funding for donor funding). While Kenya has done well in the establishment of the ‘Linda Mama’ initiative (Orangi *et al.*, 2021), which ensures free antenatal care, delivery and post-natal care services, significant gaps remain in the provision of free maternity care. Adolescent girls are subjected to informal or additional payments to access services due to lack of funding for human resources for health, commodity supplies and medical equipment, which was acknowledged as a challenge by all respondents (Banke-Thomas *et al.*, 2021).

### Socio-cultural context impacts choice of service provider

TBAs have been a controversial and contentious issue in Kenya. Debates concerning their activities in the community arose amidst high numbers of maternal deaths as a result of maternal complications (WHO, 2015). They continue to be key providers (birth companions) of maternal and neonatal health care in regions and situations where the formal health system has poor coverage or acceptability, or costs are considered to be high (Anono *et al.*, 2018). Trust is critical in the choice of a provider (Gilson, 2003); in many cases pregnant adolescents may seek out the services of a TBA due to the discrimination they face in facilities, unforeseen costs and the mistrust these create (Habib *et al.*, 2023). The value of being fed a meal post-delivery with TBAs was also highlighted by respondents. This may be particularly relevant in the context of high poverty, where many of these girls have been disowned by their families and told to fend for themselves. This impact is further exacerbated by restrictions on SRH education for adolescents and negative gendered and social norms surrounding contraception use among adolescents as highlighted by CHVs and facility service providers.

Service providers in the health facilities felt the lack of a consistent supply of essential commodities or equipment inhibited their ability to provide quality sexual reproductive health services, forcing them to refer clients for these services outside the facility. This denies adolescents’ access to services at the health facility and fuels mistrust and a lack of confidence in the health system as voiced by our respondents (Hirai *et al.*, 2020). These issues may also be compounded in informal settlements where health-care workers expressed concern for their own safety, which further limits choice of health-care provider within this context. That CHVs feel at risk when providing services within urban informal settlements has previously been reported, highlighting the importance of context in delivering services (Steege *et al.*, 2018).

Adolescent mothers often experience challenges during the intra-partum and immediate post-partum period and may be treated negatively by family and community members, which further compounds their negative experiences (Chemutai *et al.*, 2020; Erasmus *et al.*, 2020). This sentiment was equally voiced by our respondents, who reported that in the context of informal settlements high rates of gender-based violence in the community contribute to adolescent pregnancy. The pregnancy may then result in the adolescent being disowned or abandoned by their family—a situation that adds a further layer of vulnerability to these young girls (and their partners) who are still young dependants, and suddenly find themselves having to be independent providers. A lack of parental support in informal settlements may also be a risk factor for teenage pregnancy (Mumah *et al.*, 2020). The study found that parents may sometimes benefit from the transactional sex the adolescents engage in and that living conditions (families sharing one room) expose adolescents to sexual acts (among adults) from a young age. Although this was not reported in our study, it highlights how critical the unique context of urban informality is to understanding the structural drivers of adolescent pregnancy.

### Lack of attention to male partners in programming

Policy makers appreciated the limited programmes available for adolescent fathers. This both exposes adolescent girls to pregnancies, and contributes to poor health outcomes as the involvement of male partners has been shown to positively influence the health and well-being of the family and promote equitable gender norms (Daniele, 2021). Involvement of male partners is critical both for ensuring parenting is a joint responsibility and for shifting gender roles. Currently, however, the legal framework in Kenya threatens the meaningful inclusion of male partners of adolescent girls (GoK, 2006). Our findings highlight a critical gap that should be addressed to support adolescent girls, and gender-transformative change in this space. Additionally, interventions aimed at reducing teenage pregnancy must also involve males especially in patriarchal societies like Kenya if results are to be achieved.

### A way forward: moving from reproductive rights to reproductive justice for adolescents in urban informal settlements

Reproductive justice was founded on the principle that reproductive rights movements failed to meet the needs of many marginalized women (Morison, 2021). Current Kenyan policies that recognize SRH rights of adolescents fail to acknowledge the many, complex challenges facing these young people in urban informal settlements. Reproductive justice puts reproductive rights within a social justice framework—recognizing that an individual’s ability to realize their rights is shaped by social factors and social injustices. These intersecting social injustices, for example, as shown by the high rates of intimate partner violence against women, are key to pregnant adolescents in Korogocho (Ringwald *et al.*, 2022). Reproductive justice and person-centred care entails an analysis of the power structures at play, addressing the intersecting oppressions faced by adolescents and supporting the health-care providers with the resources required for adolescent-friendly and gender transformative services (Eaton and Stephens, 2020; Morison, 2021; SisterSong, 2023). This means comprehensive training, supportive supervision and continued sensitization against social norms and stigma, which have negative repercussions, as well as the need for comprehensive mental health support for this group (Kumar *et al.*, 2018). Reproductive justice also requires addressing intersecting inequities faced by pregnant and parenting adolescents in informal settlements, such as stigma, precarity, poverty, educational inequities, risks of sexual and gender-based violence, and restrictive legalities. Centering the perspectives of marginalized girls and boys via participatory approaches will enable them to share their experiences and realities (Eaton and Stephens, 2020; Morison, 2021). Meaningful engagement by adolescents in leadership, and participation in the design, implementation and evaluation of interventions is key to ensure accessibility, acceptability and quality outcomes in service delivery (WHO, 2019; George *et al.*, 2021).

Adolescents’ access to health services may be dependent on a myriad of political, social, structural, economic and environmental factors. Current and future public health interventions and policies should take account of these determinants to improve both the rights of adolescents to access quality respectful SRH services and outcomes, and to address the intergenerational, geographical and health dimensions. This requires sufficient resourcing and appropriate indicators that speak to these unique contexts. This will only be feasible if participatory approaches are explored through working with adolescents themselves (Duke, 2020) and if accountability is built into the developed policies to ensure proper implementation (Yamin and Mason, 2019).

## Study limitations

This research focused on one urban informal settlement, Korogocho, which is among the largest settlements in Kenya and the findings may not be generalizable to all informal settlements within Kenya. However, a strength is the triangulation of data across different sources, and as such the themes may be comparable in other similar settings. A key limitation is that the voices and perspectives of pregnant adolescents themselves are not included; these have been written up elsewhere (Otiso *et al*., forthcoming).

## Conclusion

Bridging the gap between policy and practice requires acknowledging and addressing the intersecting inequities that limit reproductive justice for adolescents in urban informal settlements. It also requires centring the needs and realities of vulnerable and excluded adolescents via sustainable social protection strategies, provision and training on youth-friendly and gender transformative services that include male partners, holistic care packages, and support for reintegration into their communities. Strategies also need to meet the safety and security requirements of health workers enabling them to provide care safely in these contexts.

## Supplementary Material

czad070_Supp

## Reflexivity Statement

There are five female authors with a span of extensive work and experience in implementing research informal urban settlements. The authors are of different levels of seniority; three are Kenyan (L.A.O., A.N., L.N.O.) and two are British (R.S., S.T.). The Kenyan researchers span research and health practitioner roles in Nairobi and throughout Kenya. The first and corresponding author (L.N.O.) who led on all aspects of the study is a Kenyan health systems researcher with vast experience in translating research into policy in the health system. L.N.O. was supported in study conceptualization, design and analysis, and interpretation by A.N., a researcher in Kenya with expertise in implementation research on gender equity and gender-based violence, and L.N.O. a senior health systems researcher in Kenya, with research, management and medical expertise who is engaged in policy influence and translation of research into policy with the Ministry of Health and international bodies such as WHO and Health Systems Global. R.S. also supported on study conceptualization and paper framing and writing. R.S. was involved in the study as L.A.O.’s MSc dissertation supervisor at LSTM she works on participatory and qualitative methodologies focused on health systems, community health workers, sexual and reproductive health, and gender and intersectionality. S.T. is a senior social scientist and health systems researcher with research interests and projects on strengthening health systems and equity in different contexts (including in fragility and in informal urban settlements). S.T. is L.N.O.’s mentor in the HSG’s first-time publication scheme and supported paper framing and writing.

## Data access statement

Data not currently accessible as it has not been anonymised.
