# Supplementary material for: Policy and practices shaping the delivery of health services to pregnant adolescents in informal urban settlements in Kenya
Source: Health Policy Plan. 2023 Nov 16;38(Suppl 2):ii25–35. doi: 10.1093/heapol/czad070 (PMC10666924; doi:10.1093/heapol/czad070)
Supplement: czad070_Supp [file czad070_supp.zip › suppl_data/KII Guide for National and County stakeholders- 23062021[16]RSLAO.docx]

**KII GUIDE FOR NATIONAL COUNTY AND SUB-COUNTY LEVEL PARTNERS/STAKEHOLDERS**

**Stakeholder’s Role**

1. Can you briefly tell us about yourself? ***(Explore designation and duration of service)***
2. What is your role as a National/County/Sub county focal person in promotion of maternal health service delivery of adolescent girls?
3. What are the key components of Adolescent maternal service provision policy?

**Stakeholder’s Relevance**

1. Do you think that access and utilization of maternal health services among pregnant and parenting adolescents girls is relevant in health promotion? If Yes, How? If No, Why Not?
2. What are some of the efforts national, county and Sub-county health management sector have implemented to ensure pregnant and parenting adolescent girls maternal health services are key in health promotion policy guidelines and strategies?

**Stakeholder Effectiveness/Achievements**

1. In your opinion, what strategies have the National/County/Sub-county put in place to ensure provision of pregnant and parenting adolescent girls maternal health services? ***(Probe for program modules i.e.***

- Adolescent maternal health policies
- Advocacy of adolescent maternal health services
- Access of the maternal services to adolescents in all spheres
- Improved facility capacity in terms of structure
- Improved capacity of health care workers
- Support structures in place for access and utility of maternal health services

**(Probe for reasons for responses)**

1. In your opinion, what are the barriers that the National/County/Sub-county faces in ensuring provision of pregnant and parenting adolescent girls maternal health services? ***(Probe for program modules i.e.***

- Adolescent maternal health policies
- Advocacy of adolescent maternal health services
- Access of the maternal services to adolescents in all spheres
- Improved facility capacity in terms of structure
- Improved capacity of health care workers
- Support structures put in place for access and utility of maternal health services

**(Probe for reasons for responses)**

1. Are there policies or strategies put in place to support partner involvement in adolescent male partner for maternal health service delivery? If Yes, which ones? If No, why?
2. If there are unintended but positive results from support of male partner involvement in maternal health care, what can be done to cascade the same in other health areas?
3. What are the challenges faced in provision of the adolescent maternal health service in uptake of services by beneficiaries? **(Probe for governance, financial, economic, socio-cultural** e.g. attitudes, gender considerations – SGBV, decision making of girls, access to information**, infrastructural issues)**
4. **Is data used in decision making for this group? & how is this data disaggregated? E.g. age/gender/disability?**

**Effectiveness of the policies/strategies employed in the provision of maternal health service among pregnant and parenting adolescent girls.**

1. In your opinion, *w*hat would you say about the effectiveness of the strategy applied by the National/County/Sub-county in ensuring provision of maternal health services for pregnant and parenting adolescents’ girls and their male partners? Why?
2. What will you say about how the National/County/Sub-county is working with key stakeholders especially NGOs in ensuring development of policy, capacity building, advocacy, access and provision of maternal health services for pregnant and parenting adolescents girls and their male partners? **(Probe for coordination between NGOs other partners and beneficiaries)**
3. To what degree are the viewpoints of pregnant and parenting adolescent girls and male partners incorporated into policies/strategies – what mechanisms are in place to support co-production of knowledge?

**Lessons Learned and Recommendations**

1. What are the lessons learnt from the implementation put in place for provision of maternal health services for pregnant and parenting adolescents and their male partners?
2. What are the best practices that may be adopted from the ongoing strategies?
3. What recommendations do you think should be put in place to ensure that provision of maternal health services for pregnant and parenting adolescents and their male partners is equitable, of quality and accessible?

**THANK YOU SO MUCH FOR YOUR PARTICIPATION**
